# Validation of efficacy and mechanism of Sanwei-Tanxiang powder in improving myocardial ischemia reperfusion injuries

**DOI:** 10.1038/s41598-020-80861-6

**Published:** 2021-01-12

**Authors:** Yu-Hui Sun, Ren Bu, Yue-Wu Wang, Yu-Chong Hu, Xu-Mei Wang, Xin Dong, Wen Zu, Yan Niu, Peng-Wei Zhao, Peng Sun, Shi-Hang Ru, Jing-Kun Lu, Sheng-Sang Na

**Affiliations:** 1 Department of Pharmacy, Chifeng Municipal Hospital, Chifeng, China; 2 School of Pharmacy, Inner Mongolia Medical University, Huhehot, China; 3 Center for New Drug Safety Evaluation and Research, Inner Mongolia Medical University, Huhehot, China; 4 Inner Mongolia Autonomous Region People’s Hospital, Huhehot, China; 5 Inner Mongolia Medical University, Huhehot, China; 6 School of Basic Medicine, Inner Mongolia Medical University, Huhehot, China; 7 Radiotherapy Department, Chifeng Municipal Hospital, Chifeng, China; 8 Institute of Mongolian Medicine, Inner Mongolia Medical University, Huhehot, China

**Keywords:** Biotechnology, Drug discovery, Molecular biology, Systems biology

## Abstract

Sanwei-Tanxiang powder (SWTX), a traditional Mongolian and Tibetan medicine containing a cocktail of active molecules, relieves angina pectoris and improves recovery in patients with coronary heart disease (CHD). The pharmacological effect of SWTX on CHD was analyzed at a systemic point of view in our previous studies. The bioinformatics prediction showed that the PI3K/Akt/FoxO3a pathway was one of important pathways of SWTX on treatment of coronary heart disease. Based on it, the aim of this study was to evaluate the benefits of SWTX in acute myocardial ischemic-reperfused (MIR) rat in vivo and H9c2 cardiomyoblast cells under oxidative stress induced by H_2_O_2_ in vitro, and further investigate the involvement of PI3K/Akt/FoxO3a pathway in these processes. Ex vivo, under physiological conditions, SWTX did not show any modification in the heart rate and contraction amplitude. However, against a MIR injury, SWTX pretreatment provided significant protection, including reduced ST-segment elevation, pathological changes and myocardial infarct size in vivo, meanwhile, some monomers of SWTX showed antioxidant capacity and inhibited cardiomyocytic apoptosis in vitro. The effect was correlated with the activation of the PI3K/Akt/FoxO3a signaling pathway downstream and the regulation of downstream pro-apoptotic Bim of FoxO3a experimental verified by qRT-PCR, Western blot and immunofluorescent assay. In vitro, blocking Akt and p-FoxO3a activation with the PI3K inhibitor LY294002 effectively suppressed the protective effects of several active monomers (including quercetin, macelignan,methyleugenol and Santol) of SWTX against H_2_O_2_-induced injury. Collectively, these results suggest that SWTX decreases I/R injury, and the PI3K/Akt/FoxO3a pathway takes part in protection during this process, gallogen (G3) and quercetin (G8) of GZ, methyleugenol (R2) and macelignan (R7) of RDK, santol (T1) of TX are responsible at least in part for SWTX’s cardioprotection effect.

## Introduction

Myocardial infarction (MI) is one of the leading causes of death for adults worldwide. Modern treatment aiming at the most serious MI, ST elevation MI, involves primary percutaneous coronary intervention (PPCI), thrombolytic therapy and angioplasty. Although reperfusion potentially allows salvage of viable ischemic myocardium, in fact, it comes at a cost as it can confer ischemia/reperfusion (I/R) injury (MIRI). The damage involves arrhythmia, expansion of infarct size, loss of mitochondrial calcium homeostasis, dysfunction of microcirculation, influx of inflammatory cells, edema and apoptotic processes^1–4^. The damage attributable to MIRI is estimated between 25 and 50% of the total infarct^1^. Therefore, novel therapeutic medicine is urgently required to limit the extent of MIRI after MI.

The ancient Sanwei-Tanxiang powder (SWTX) was first recorded in “The Four Medical Classic” in the eighth century, and has been commonly used to improve heart function in CHD clinical practice of Mongolian and Tibetan medicine for a long time^5,6^. Based on the principle of “Jun-Chen-Zuo-Shi” (“emperor-minister-adjuvant-courier”) in traditional medicine, SWTX consists of GuangZao (*Fructus Choerospondiatis*, emperor, GZ), RouDoukou (*Nutmeg*, minister, RDK) and Tanxiang (*Santalum album L.*, adjuvant, TX), and the three raw herbs were mixed in a ratio of 1:1:1. Our previous studies have demonstrated that GuangZao and RouDoukou in combination exert anti-myocardial ischemia effects by regulating stress and inflammatory responses, ameliorating cardiomyocyte apoptosis in vivo^7^. In recent years, systems pharmacology has received much attention on the drug discovery and clinical practice of complex diseases, therefore, we had used this novel strategy to identify the active compounds of SWTX and their therapeutic targets in previous studies^8^. The results revealed that the PI3K/Akt/FoxO3a pathway was possibly one of crucial pathways of SWTX on treatment of CHD, and quercetin, gallogen, macelignan, methylisoeugenol, santol and others could be active ingredients. Based on it, the objective of the present study was to determine the anti-MIRI property of SWTX. The effects of of SWTX on gene and protein expression profiles of PI3K/Akt/FoxO3a pathway were studied using rat myocardial ischemic-reperfused (MIR) model in vivo. H9c2 cardiomyoblast cells induced by H_2_O_2_ in vitro were used to explore the relationship between the active compounds of SWTX and PI3K/Akt/FoxO3a pathway as cardioprotective agents.

## Materials and methods

### Drug preparation

GuangZao (GZ), RouDoukou (RDK) and Tanxiang (TX) were purchased from Bezhou Wanshixiang Herbal Pieces Co.Ltd. All the botanical characters of the herbs were identified by Prof. Junchan Qiao (Inner Mongolia Medical University). Quercetin, gallogen, macelignan, Santol and other monomers (purity ≥ 98%) were purchased from J&K Scientific Ltd. (Beijing, China). GZ was extracted in a ten-fold volume of 70% ethyl alcohol by reflux for three times, 2 h one time, and then the extract solution was dried to a powder. The essential oil and butter from RDK and the TX volatile oil were obtained with a supercritical CO_2_ extraction apparatus, which had worked at 35–36 MPa and 35 °C for 2 h. Extraction yield of GZ, RDK and TX was 20.0%, 6.0% and 3.0% respectively.

### Reagents

Antibodies included rabbit anti-Akt1 (1:1000, CST: #75,692, USA), rabbit anti-p-Akt1 (1:1000, CST: #9018, USA), rabbit anti-FoxO3a (1:1000, CST: #2497, USA), rabbit anti-p-FoxO3a (1:1000, CST: #9466, USA), rabbit anti-Bim (1:1000, CST: #2933, USA), rabbit anti-GAPDH (1:1000, CST: #2118, USA) and goat anti-rabbit secondary antibody (1:3000, Bioss biotechnology Co. LTD., China). Others included Total RNA isolation kit (Tiangen Biotech Co., LTD., China), TRIzol reagent Kit, SYBR Premix Ex Taq II enzyme kit, and PrimeScriptRT Master Mix (Takara Bio, Inc., China), Evans blue, 2,3,5-triphenyltetrazolium chloride (TTC, Solarbio Technology, China), LY294002 (PI3K inhibitor, Sigma), Lactate dehydrogenase (LDH) and Creatine kinase (CK) Kits (MedicalSystem Biotechnology CO. Ltd, China), goat anti-rabbit IgG/FITC (Beijing biosynthesis biotechnology CO.Ltd, China), Annexin V-FITC Apoptosis Detection Kit(Beyotime biotechnology, China) and BCA protein Assay kit (Merck Millipore Technology, Germany). Oligo synthesis was performed by Sangon Biotech co. LTD (Shanghai, China).

### Statement

(1) All experiments, including methods and operations were approved by the Animal Care and Ethics Committee of Inner Mongolia Medical University, China. (2) All experimental methods were performed in accordance with guidelines and regulations of the Ethics Committee of Inner Mongolia Medical University, China.

### Rat model of myocardial ischemia/reperfusion (MI/R)

All experiments were conducted on Male Sprague–Dawley rats (Experimental Animal Center of Inner Mongolia Medical University, China) at 6–8 weeks of age. Rats were randomized into eight groups, including GZ, GZ + RDK and SWTX group (*n* = 6); control (sham one), model, GZ + model, GZ + RDK + model and SWTX + model group (*n* = 15). The sham one and the model group received 0.3% sodium carboxymethyl cellulose (CMC-Na) solution (2 mL/kg/day, i.g.). The herb groups and herb + model groups each received GZ extract (360 mg/kg/day, i.g.), GZ extract (360 mg/kg/day, i.g.) + RDK extract (108 mg/kg/day, i.g.), GZ extract (360 mg/kg/day, i.g.) + RDK extract (108 mg/kg/day, i.g.) + TX extract (54 mg/kg/day, i.g.) in accordance with the extraction rate of each herb and fivefold clinical equivalent dose of SWTX. Each group was administered for a period of 10 days and the model and herb + model groups induced MI/R model 1 h after the last dose.

The rats were anesthetized using with 10% chloral hydrate (400 mg/kg body weight, i.p.) and ventilated with air using a small-animal respirator (Chengdu Technology &Market Co. Ltd., China) (tidal volume, 2 ml/100 g; ventilator frequency, 60 breaths/min). The BL-420 biological function experimental system (Thai union technology Co. Ltd., China) was placed to record electrocardiogram (ECG), and a homothermal equipment was used to maintain rats in warm. The heart was exposed via a left thoracotomy, and the left anterior descending (LAD) coronary artery was ligated for 30 min followed by a 2 h reperfusion. Coronary arterial occlusion was confirmed by colour change of the left ventricle. The same procedure was performed for sham-operated group rats except no ligation.

At the end of the experimental period, blood was collected via abdominal aorta and serum samples were subsequently stored at − 80 °C for biochemical assays. The heart tissue was excised and rinsed immediately in ice-cold normal saline, then stored at − 20 °C or − 80 °C for further analysis.

### Assay of serum myocardial injury markers

Creatine kinase-MB (CK-MB) and lactate dehydrogenase (LDH) in serum were measured by Biochemical analyzer (Biobase-Sapphire, Ireland).

### Infarct size determination and histopathology

After blood collection, the LAD was ligated again in situ, and 2% Evans blue 2–3 ml was injected into the internal jugular vein. The rat hearts blue stained were cleaned in phosphate buffer solution (PBS) for three times and stored at − 20 °C for more than 2 h, then were sliced into six sections along the long axis. The sections were incubated in 2% TTC (dissolved in PBS) for 20 min at 37 °C and fixed in 10% formalin overnight. The infarct size (IS) was calculated as described by Zhang^9^. Briefly, images were captured and the area was adjusted for weight. The area at risk (AAR) was the part no dyed blue by Evans blue in the section of left ventricle. The infarct area located in the pale part was no dyed by TTC.

To evaluate the extent of ischemia and infarct, the heart tissue were sectioned (5 μm), stained with hematoxylin /eosin (HE). The details were described in our report ^7^.

### Cell viability

H9c2cells were purchased from CAS (Shanghai Institutes for Biological Sciences, China),and were cultured in Dulbecco's Modified Eagle Medium(DMEM) with 10% fetal bovine serum (FBS, PAN-Biotech, Germany), 1% penicillin–streptomycin in a 5% CO_2_ incubator at 37 °C. H9c2cells were seeded in 96-well plates with a concentration of 1 × 10^4^cells per well. After incubated at 37 °C for 24 h, the cells were treated with different experimental settings: (1) vehicle control (0.1% DMSO/PBS); (2) the active compounds of SWTX respectively; (3) model (900 μM H_2_O_2_ for 22 h); (4) the same settings as in 2, but with 900 μM H_2_O_2_ for 22 h; (5) the same settings as in 4, but quercetin, gallogen, macelignan, methyleugenol and Santol group with 50 nM LY294002. The detailed information of these predicted active monomers of SWTX was listed in Supplementary Table S1. After 24 h incubation, 10 μl 3-(4,5-dimethylthiazol-2-yl)-2,5-diphenyltetrazolium bromide (MTT) solution was added into each well, and continued for 4 h. Subsequently, the formazan crystals were dissolved with 100ul DMSO per well, and the plates were measured by a microplate reader (Thermo multiskan FC, Finland) at 490 nm.

### Flow cytometry

Apoptotic cells were quantified by flow cytometry. Briefly, the cells were seeded in 6-well plates (2 × 10^5^ cells/ well) for 24 h and were pre-treated as indicated above. The cells were washed thrice with ice-chilled PBS and resuspended with 100 μl Annexin V Binding Buffer. Forthermore, the cell suspension was stained with annexin V-FITC and PI for 15 min in dark. Apoptotic analysis was immediately performed on a flow cytometer FACS Aria II (BD Biosciences, San Jose, USA).

### Quantitative real-time PCR

qRT-PCR was performed on a Step-One Plus (Applied Biosystems). GAPDH was used to normalize the gene expression. The following primer sequences were used: GAPDH: forward 5′-CGGCAAGTTCAACGGCACAG-3′, reverse 5′-GACGCCAGTAGACTCCACGACAT-3′; Akt1: forward 5′- ATGAACGACGTAGCCATTGTGAAGG -3′, reverse 5′-CCTTCACAATGGCTACGTCGTTCAT -3′; PIK3R1: forward 5′-TGGCATCTTCTCCTTCCAGCCT-3′, reverse 5′-GGGGCAGTGTTTGCAGGTTATGCAT -3′; Bim: forward 5′- CGGATCGGAGACGAGTTCAATGAG -3′, reverse 5′-CCAGACCAGACGGAAGATGAATCG -3′;

### Western blotting

The left ventricle ischemic tissue samples or cells samples were homogenized with RIPA buffer (1% PMSF) for 30 min. In total protein extract, protein expressions ofAkt,p-Akt, FoxO3a, p-FoxO3a, Bim and GAPDH were determined by western blot which was performed as described previously^7^.

### Immunofluorescence staining of FoxO3a

H9c2 was grown in chamber glass slide. Cells were fixed for 15 min in cold 16% methyl aldehyde in PBS. After washing with PBS for three times, the cells were incubated with 5% goat serum and 0.3% TritonX-100 in PBS for 60 min at room temperature and incubated with goat IgG as a negative control or rabbit anti-FoxO3a (diluted 1:200) in blocking buffer at 4 °C overnight. After washing three times, the cells were incubated with goat anti-rabbit IgG/FITC (diluted 1:500) for 1.5 h, washed three times and reacted with DAPI (4′,6-diamidino-2-phenylindole). Cells were examined using Laicamicroscope (DFC450 C, Germany).

### Statistical analyses

Data are expressed as mean ± SD. One-way ANOVA was used to determined statistical differences. For statistical comparision of means between two groups, unpaired two-tailed Student’s *t*-test was carried out using Graph Pad Prism 6.0 software, and *p* < 0.05 were considered significant.

## Results and discussion

### SWTX decreased myocardial injury and improved cardiac function in MI/R rats

**Figure 1:**
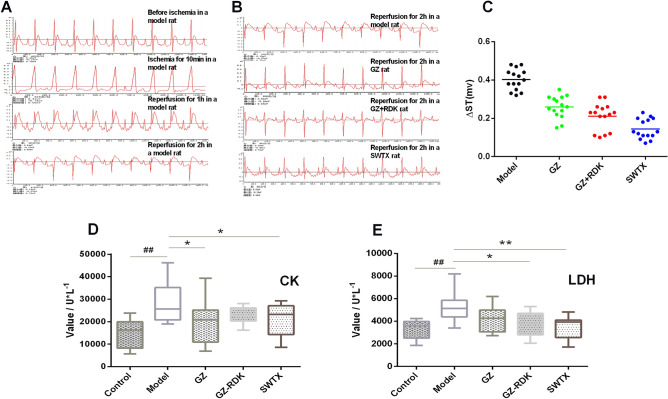
SWTX treatment reduced the severity of myocardial ischemia. (**A**) The typical electrocardiograms (ECG) of model rats at each node. (**B**) The typical electrocardiograms (ECG) of treatment groups after reperfusion for 2 h. (**C**) ST alteration value of each group (∆ST). (**D**) The level of LDH. (**E**) The level of CK. Values are mean ± SD (*n* = 15 in each group). ^##^*P* < 0.01 versus control group; **P < 0.01 versus model; *P < 0.05.

Clinical application for long time has demonstrated that SWTX was safe in humans. This study also demonstrated GZ, RDK, SWTX administration didn’t disturb the basic cardiac physiological parameters (Supplementary Figure S1). Our previous study revealed attenuation of myocardial injury in isoproterenol-induced myocardial ischemia animal model by the combined application of GZ and RDK^7^. In order to evaluate the cardioprotective effects of SWTX in vivo, a rat model myocardial ischemia–reperfusion injury was used. As illustrated in Fig. 1A,C, rats were subjected to 30 min of ischemia followed by 2 h of reperfusion, the ECG patterns of model rats revealed that variation of ST-segment markedly upward (model group, 0.42 ± 0.05). After 2 h of reperfusion, the ECG exhibited the reduction of myocardial ischemia with varying degrees in the GZ, GZ + RDK and SWTX groups compared with the model group (GZ, GZ + RDK or SWTX*vs.* model*, P* < 0.01, Fig. 1B,C). In addition, SWTX (0.14 ± 0.05) was observed with a more significant effect compared with GZ (0.26 ± 0.05) or GZ + RDK (0.21 ± 0.07) (SWTX *vs.* GZ or GZ + RDK, *P* < 0.01 or *P* < 0.05).

To assess the effect of SWTX in myocardial injury after myocardial infarction, serum cardiac markers (LDH and CK), myocardial infarct size, and histological changes were measured. As is shown in Fig. 1D,E, the CK, LDH content is 1.9 or 1.6 folds higher in I/R model group than in the control group (CK, 14,834.0 ± 6494.1 U/L *vs.* 28,464.9 ± 9729.7 U/L, *P* < 0.01; LDH, 3273.1 ± 890.5 U/L *vs.* 5303.0 ± 1291.0 U/L, *P* < 0.01) (control *vs.* model group). In contrast, SWTX pretreatments were found to significantly reduce the I/R-induced leakage of LDH and CK compared to model rats (CK, 21,021.3 ± 7326.0 U/L, *P* < 0.05; LDH, 3553.3 ± 959.5 U/L, *P* < 0.01) (SWTX *vs.* model). Meanwhile, GZ significantly decreased CK (CK, 19,294.3 ± 9262.4 U/L, *P* < 0.05), and GZ + RDK reduced LDH (LDH, 3835.9 ± 1150.8 U/L, *P* < 0.01). There was no significant difference between GZ, GZ + RDK and SWTX groups (GZ or GZ + RDK *vs.* SWTX, *P* > 0.05).

**Figure 2:**
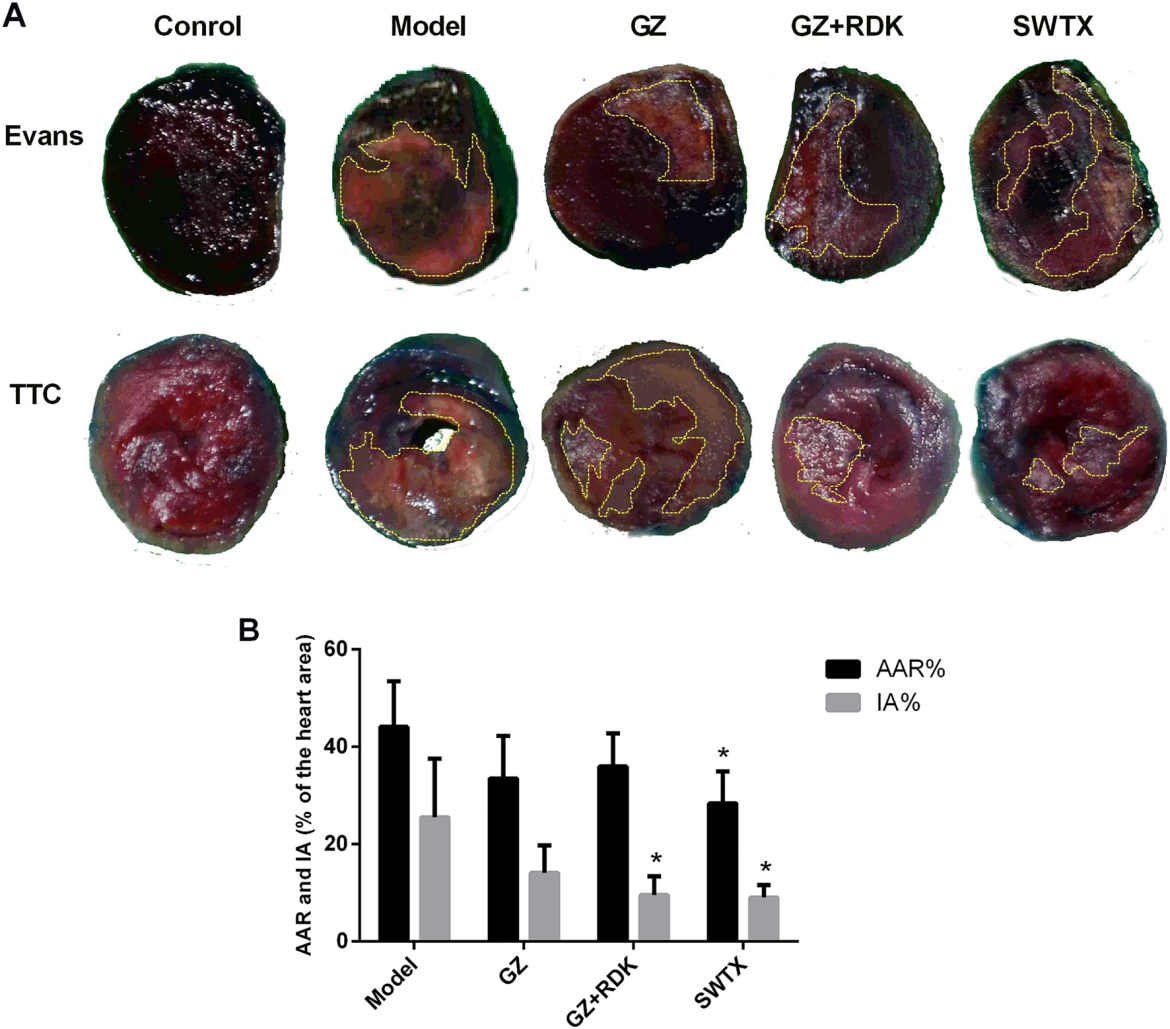
Representative samples stained by Evans blue or triphenyl tetrazolium chloride (TTC). (**A**) SWTX treatment reduced myocardial infarct size (IA) and histological changes. The area at risk (AAR) was the part un-dyed blue by Evans blue and the myocardial infarct size (IA) was measured by TTC unstaining. (**B**) The bar graph showing cumulative data of AAR and IR. Values are mean ± SD (*n* = 5 in each group). **P < 0.01 versus model; *P < 0.05.

In I/R model group, the area at risk (AAR or RR) un-dyed by Evans blue and the infarct area (IA) un-stained by TTC (shown in pale) accounted for respectively 44.08 ± 9.33% and 25.50 ± 12.07% of heart. Compared with the model group, pretreatment with GZ + RDK and SWTX was found to decrease AAR and IA (Fig. 2).

**Figure 3:**
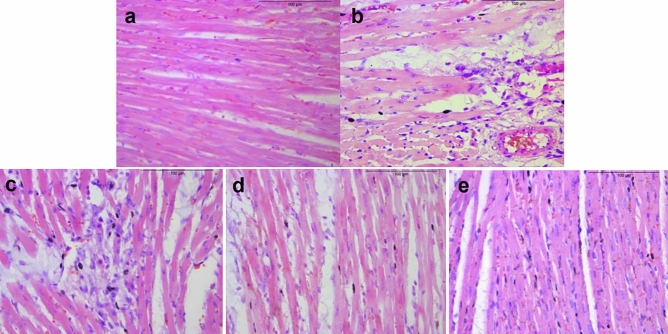
Histopathological observations of the heart (200 ×). (**A**) Control rat heart revealed normal architecture of myocardium. (**B**) The I/R model rats showedfragmentation of heart myofibrils, necrosis, vacuolar degeneration, loss of striations, hyperemia, and inflammatory infiltration. (**C**) GZ-treated rats showed inflammatory infiltration. (**D**) GZ + RDK-treated rats showedpartly vacuolar degeneration, fragmentation of heart myofibrils, loss of striations, (**E**) SWTX-treated rats showedwider gaps between myocardial fibers, no inflammatory infiltration. *n* = 3 in each group.

Likewise, Ischemia–reperfusion led to significant histopathology in cardiac sections stained with HE, which was visible as extensive disruption and fragmentation of heart myofibrils, loss of striations, hyperemia, and inflammatory infiltration in the model rats.Administration of GZ, GZ + RDK or SWTX all reduced the myocardial necrosis in different degrees. The heart tissue of GZ, GZ + RDK treated rats respectively showed partly vacuolar degeneration, mild neutrophil infiltration, or wider gaps between myocardial fibers. Meanwhile, SWTX group showed better cardioprotection because of the relatively intact myocardial fibers (Fig. 3).

These combined results demonstrate that SWTX inhibited I/R injury by reducing ST-segment elevation, pathological changes and myocardial infarct size and improving cardiac function in vivo, and three herbs, GZ, RDK and TX, had synergistic effects.

### The active monomers of SWTX exerted effect against H_2_O_2_-induced oxidative injury on H9c2 cells

**Figure 4:**
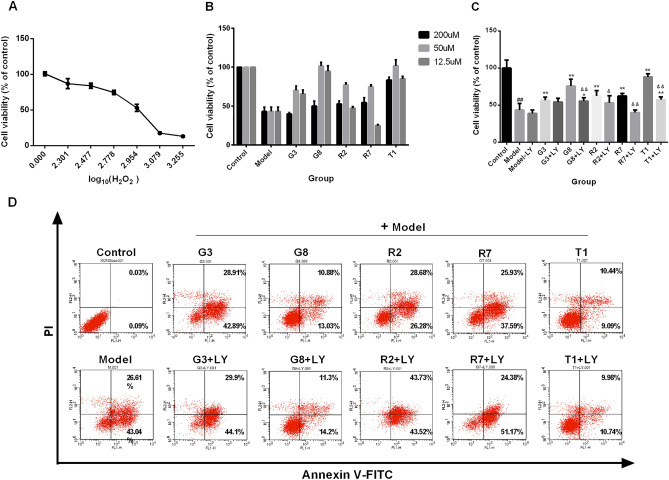
Effects of the activemonomers of SWTX on H_2_O_2_-induced H9c2 cell oxidative injury. (**A**) H9c2 cells treated with 200–1800 μΜ H_2_O_2_ for 22 h, shown marked reductions with increasing dose in cell viability (*n* = 6). (**B**) 12.5, 50, 200 μΜ G3, G8, R3, R5 and T1 pretreated H9c2 cell for 2 h and co-incubated with 900 μΜ H_2_O_2_ for additional 22 h, significantly improved H9c2 cell viability in comparison to the cells singly treated with 900 μΜ H_2_O_2_, among them, G8 and T1 shown best (*n* = 6). (**C**) The protective ability of 50μΜ G8, R3, R5 and T1 groups is significantly reduced by PI3K inhibitor LY294002 (50 nM) in H9c2 cell (*n* = 6). (**D**) The cell apoptosis ratio was detected by flow cytometry, and the data indicated a decreasing percentage of cells in the lower and upper right quadrants (early and late stage apoptosis) in 50 μM G3, G8, R2, R7 and T1 groups compared with 900 μΜ H_2_O_2_ group, and an increasing percentage of the apoptosis cells following LY294002 (50 nM) treatment . ^##^*P* < 0.01 versus control group; **P < 0.01 versus model; *P < 0.05 versus model; ^&&^*P* < 0.01 monomer versus LY294002 + monomer and ^&^*P* < 0.05 monomer versus LY294002 + monomer.

Based on the previous results of systems pharmacology^7^ and literatures^10–16^, we investigated the cytotoxicity of the part of predicted active monomers of SWTX and their effect against H_2_O_2_ stimulus on H9c2 cells (Supplementary Table S2 and Figure S2). Compound G2 (citric acid), G4 (gallic acid), R1 (eugenol) exhibited cytotoxicity to H9c2 cells in concentration range 12.5–200 μΜ, and the others didn’t affect cell viability in 50 μΜ. Besides that, 12.5, 50 μΜ G3 (gallogen), G8 (quercetin), R3 (methyleugenol), R7 (macelignan), T1 (santol) showed proliferative capacity to H9c2. Secondly, cells were treated with increasing doses (200–1800 μΜ) of H_2_O_2_ for 22 h, as shown in Fig. 4A, H2O2 impaired cell viability in a concentration-dependent manner with IC_50_ values of 916 μΜ. Based on this, we chose the level of H_2_O_2_ 900 μM in our subsequent experiments. In order to determine the effects of the monomers on H_2_O_2_-induced cell injury, the H9c2 cells were pretreated with 12.5, 50, 200 μΜ compounds for 2 h, after which co-incubated with 900 μΜ H_2_O_2_ for additional 22 h. A majority of G3 (gallogen) and G8 (quercetin) of GZ, R2 (methyleugenol) and R7 (macelignan) of RDK, T1 (santol) of TX in 50μΜ revealed the better cardioprotection, among them, G8 and T1 showed best. However, PI3K inhibitor LY294002 (50 nM) damaged the properties of these compounds, as shown in Fig. 4B,C.

As we know, ischemia can cause cell necrosis or apoptosis in cardiomyocytes^3^. To clarify whether G3, G8, R2, R5 and T1 protect H9c2 cells against apoptosis, we analyzed apoptotic ratio of the cells co-treated with H_2_O_2_ and these compounds for 22 h by flow cytometry. The results confirmed that 50 μM G3, G8, R2, R7 and T1 efficiently reduced the apoptosis ratio from 69.65% (H_2_O_2_-treated) to 63.8%, 23.91%, 54.96%, 63.52% and 19.53%, and LY294002 (50 nM) reversed the cardioprotection of these compounds and a greater apoptosis were revealed in cells receiving LY294002 + monomer compared with cells treated with the corresponding monomer, as shown in Fig. 4C,D.

The total flavonoids of GZ were reported for preventive effects against proliferation of rat cardiac fibroblasts (CFs) induced by angiotensin II and I/R injury in rats^17,18^, and major organic acids in GZ, had cardioprotection on I/R rats^10^, as well as the essential oils of RDK is proposed to have antioxidant and antiangiogenic activities^19^, and the essential oils and principle component (santol) of TX has neuroprotective and geroprotective activities^11^. G3 (gallogen) and G8 (quercetin) of GZ, R2 (methyleugenol) and R7 (macelignan) of RDK, T1 (santol) of TX are constituents migrating to blood, major or active components in SWTX^11–13^. The literatures^11,14–16^ reports that these compounds display protective effects against oxidative stress and/or inflammation. Our result also shows that G1 (citric acid), G3, G8, R2, R7 and T1 inhibited cell injury and apoptosis induced by H_2_O_2_, in line with the reports. However, G1, G4 (gallic acid) had cytotoxicity to normal cells, and G4 didn’t show antioxidant activity, which is contradictory with Dong’s report^20^.

### PI3K/Akt/FoxO3a pathway is involved in acardioprotective action of SWTX

Studies have demonstrated that the PI3K-Akt pathway regulates cardiomyocyte size, cellularstress response, cellular antioxidant defense, inflammation and angiogenesis in both physiological and pathological condition^21,22^. The Bcl-2 family protein Bim triggers mitochondrial apoptosis, and the PI3K/Aktpathway inhibits apoptosis associated with mitochondrial pathway^23^. When cells exposed to various stressful stimuli, reduction of FoxO3a phosphorylation mediated by Akt promotes FoxO3a translocation from the cytoplasm to the nucleus, further leading to Bim which transcriptional activation dependent on FoxO3a culminates into induction of apoptosis^24^. Early observations suggested that Bim can directly activate Bax and Bak, initiating cytochrome c release as well as inhibiting the anti-apoptotic Bcl-2 proteins^25^, but recently an increasing number of studies had reported that FOXO3a only regulated Bim at mRNA levels at least in hematopoietic cells^26^, and Bim levels were inversely correlated with the induction of Bim-dependent apoptosis in myeloid and lymphoid cells^27,28^. Prafull et al.'s studies^29^ further confirmed thatBim surprisingly bound only anti-apoptotic Mcl-1 with the participation of dynein light chain 1 (DLC1) at endogenous levels, but not bound Bcl-2 or Bcl-XL, recruiting only Mcl-1 into large complexes at the mitochondrial outer membrane, and this large complexes has a higher proapoptotic activity than wild-type Bim (single Bim) in all cell lines that they tested.

**Figure 5:**
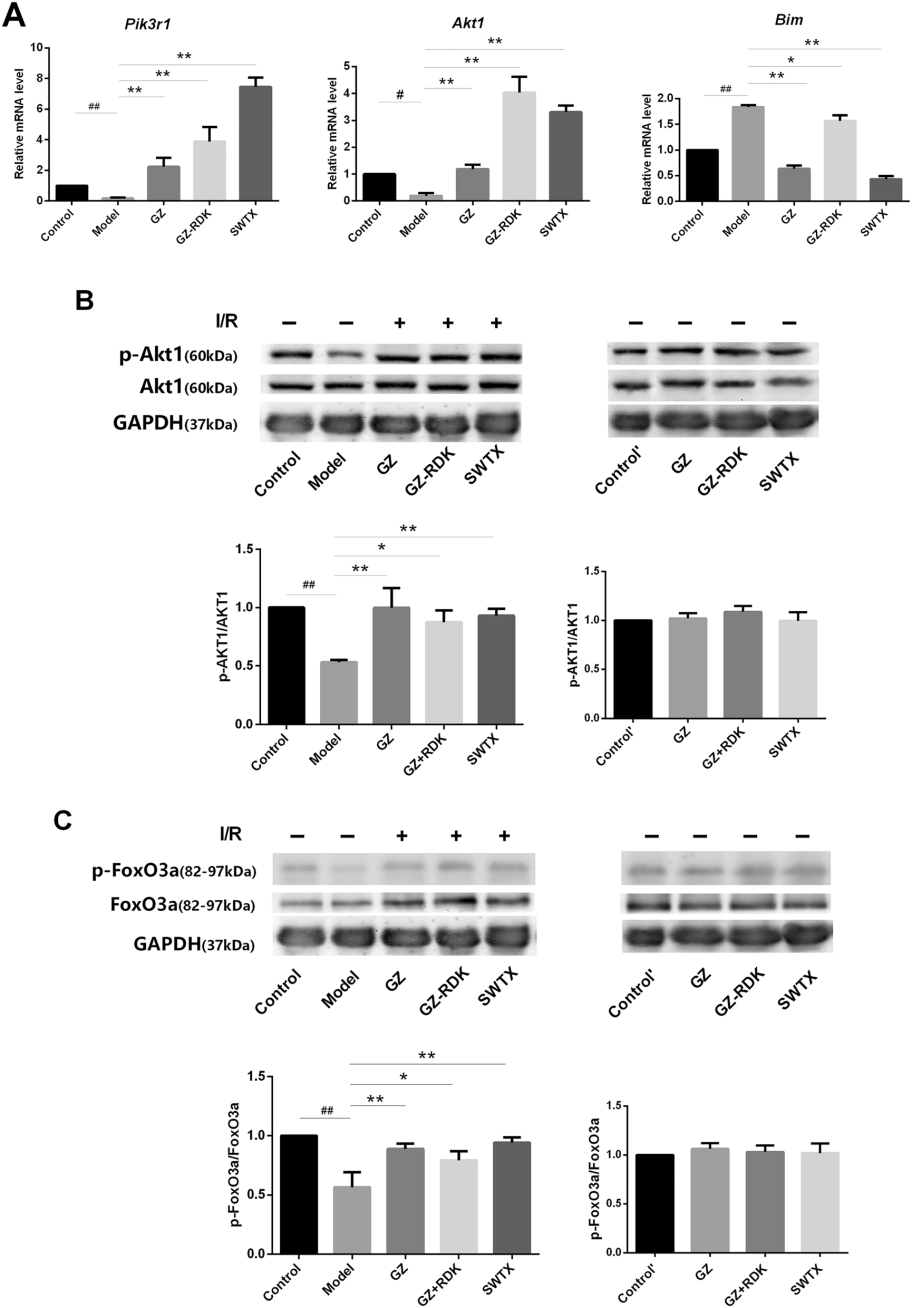

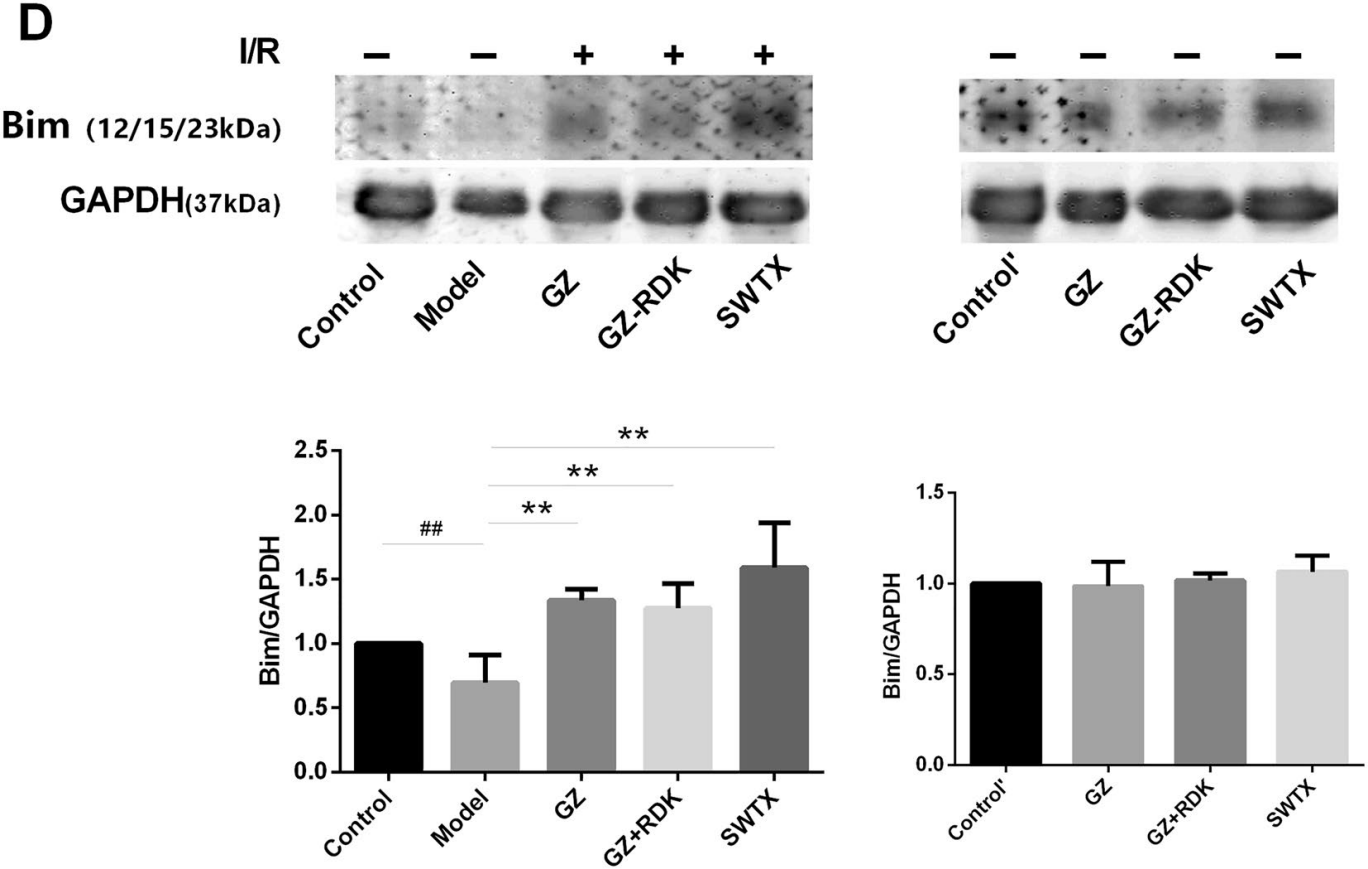
PI3K/Akt/FoxO3a pathway is involved in a protective action of SWTX on rat MI/R injury. (**A**) The mRNA levels for *Pik3r1*, *Akt, Bim* were assayed by qRT-PCR in cardiac tissues. (**B**–**D**) The representative Western blots shown the effects of SWTX on the expression of Akt, p-Akt, FoxO3a, p-FoxO3a, Bim, and GAPDH was used as loading control. Data are expressed as the mean value ± SD (*n* = 3 mice per group). ^##^*P* < 0.01 *versus* control group. *P < 0.05, **P < 0.01, versus model.

Our previous bioinformatics studies have revealed that PI3K/Akt/FoxO3a pathway was possibly one of important pathways of SWTX on treatment of coronary heart disease^8^. To confirm this finding, we analyzed SWTX on the expression of the genes of*Pik3r1*, *Akt, Bim* in rat myocardial tissues following I/R injury. As shown in Fig. 5A, the *Pik3r1* and *Akt* gene was down-regulated significantly by I/R injury in vivo, meanwhile *Bim* gene was up-regulated, which were efficiently reversed by GZ, GZ + RDK and SWTX, SWTX has highest advantages. Further, the protein expression of p-Akt, p-FoxO3a and Bim was significantly decreased in the myocardial tissue of I/R rats (*P* < 0.01 or *P* < 0.001, model *vs.* control). The reduction in Bim protein expression of the injured myocardium may be due to the formation of the large complexes (Bim- DLC1- Mcl-1) with stronger pro-apoptotic activity, although in Bim-dependent apoptosis. The altered protein expression was close to normalized in SWTX group and, to a greater extent, in GZ or GZ + RDK groups. Meanwhile, each drug didn’t disturb the protein expressions of p-Akt, p-FoxO3a and Bim in normal rats. The results were shown in Fig. 5B–D.

**Figure 6:**
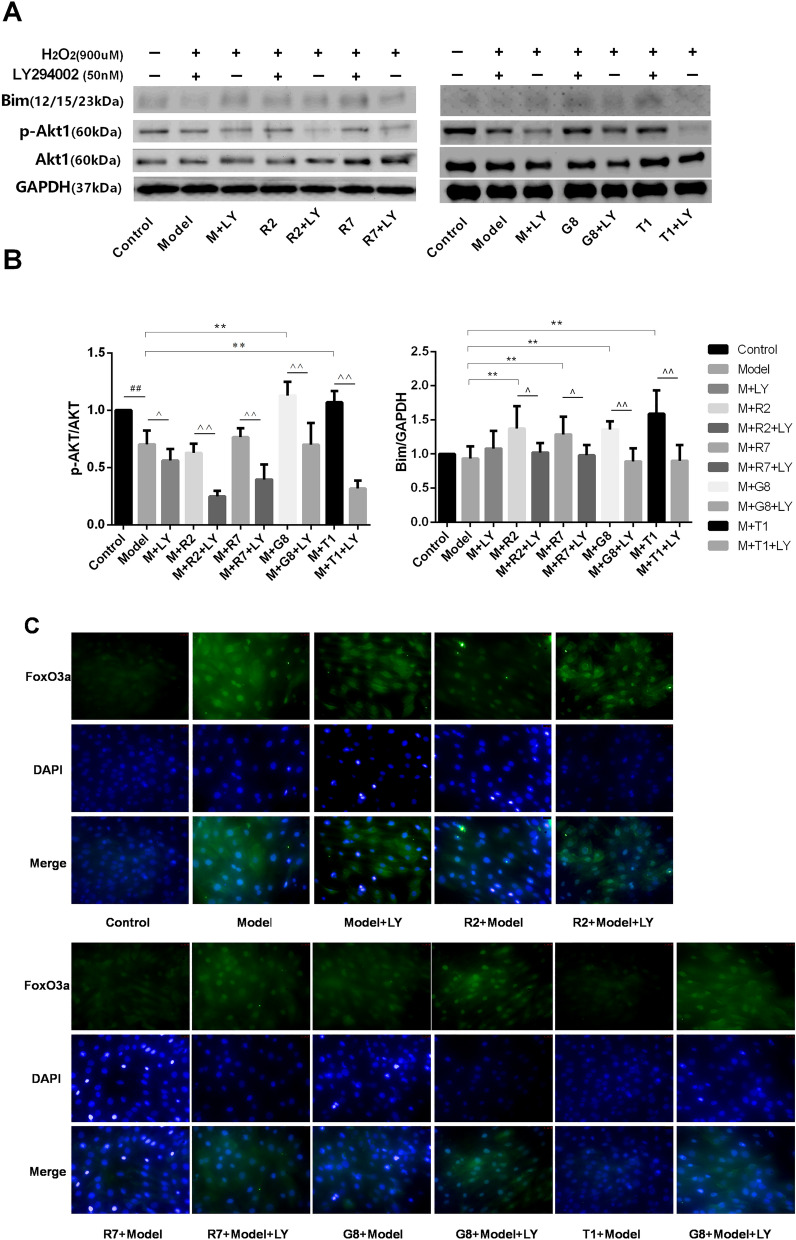
PI3K/Akt/FoxO3a pathway is involved in a protective action of representative monomers G3, G8, R2, R7 and T1 of SWTX on H9c2 cells exposed to H_2_O_2_ (900 μM) for 22 h, and PI3K inhibitor LY294002(LY, 50 nM) was used to further confirm the effects of SWTX on PI3k/Akt/FoxO3a signaling pathway. (**A**) Representative Western blots shown the effects of G3, G8, R2, R7 and T1 on the expression of Akt, p-Akt, Bim, and the interfering effects of LY294002 (LY), while GAPDH was used as loading control. (**B**) The expression of p-Akt, Akt in H9c2 cells was essayed by western blotting using a Image J analyzer. (**C**) Representative Immunofluorescence staining shown the effects of G3, G8, R2, R7 and T1 on the translocating level of FoxO3a to nucleus on H9c2 cells was essayed by Immunofluorescence staining. Data are expressed as the mean value ± SD from three independent experiments. ^##^*P* < 0.01 versus control group, *P < 0.05, **P < 0.01, versus model, ^*P* < 0.05, ^^*P* < 0.01, the monomer versus corresponding monomer + LY294002 (LY).

We previously demonstrated that PI3K inhibitor LY294002 (LY, 50 nM) blocked the cardioprotection of G8, R2, R7 and T1, which are the major compounds in SWTX. In order to understand the cardioprotection mechanisms of SWTX, we investigated the effect of representative monomers G8, R7, R5 and T1 of SWTX on the protein expression of Akt, p-Akt, Bim and the translocating level of FoxO3a to nucleus on H9c2 cells. The results were indicated in the Fig. 6. We found that H_2_O_2_ treatment at a concentration of 900 μM for 22 h significantly decreased the protein expression of P-Akt, and the nucleus level of FoxO3a compared with the control group, but not interfere with Bim. The decreasing of p-Akt induced by H_2_O_2_ was efficiently returned by G8 and T1 (50uM), but the other two (R5 and R7) did not. Bim of each monomer group was significantly higher than the H_2_O_2_ group, meanwhile, the nucleus level of FoxO3a showed the opposite trend, there was no significant difference between the monomer groups. At a concentration of 50 nM, PI3K inhibitor LY294002 suppressed significantly the protein expression of p-Akt and further promoted FoxO3a translocation from the cytoplasm to the nucleus. Responding to this case, Bim tended to increase but no significant difference compared with the H_2_O_2_ model. At the same time, LY294002 totally or partly reversed effects of G8, R7, R5 and T1 and suppressed significantly p-Akt and Bim compared with their corresponding groups. Therefore, these results further proposed that reduction of phosphorylation levels of Akt, FoxO3a was the important steps for I/R injury, SWTX and representative monomers reduced the damage via promoting phosphorylation of Akt and FoxO3a and increasing single Bim which maybe decrease the formation of the Bim- DLC1- Mcl-1 complexes with stronger pro-apoptotic activity.

## Conclusions

**Figure 7:**
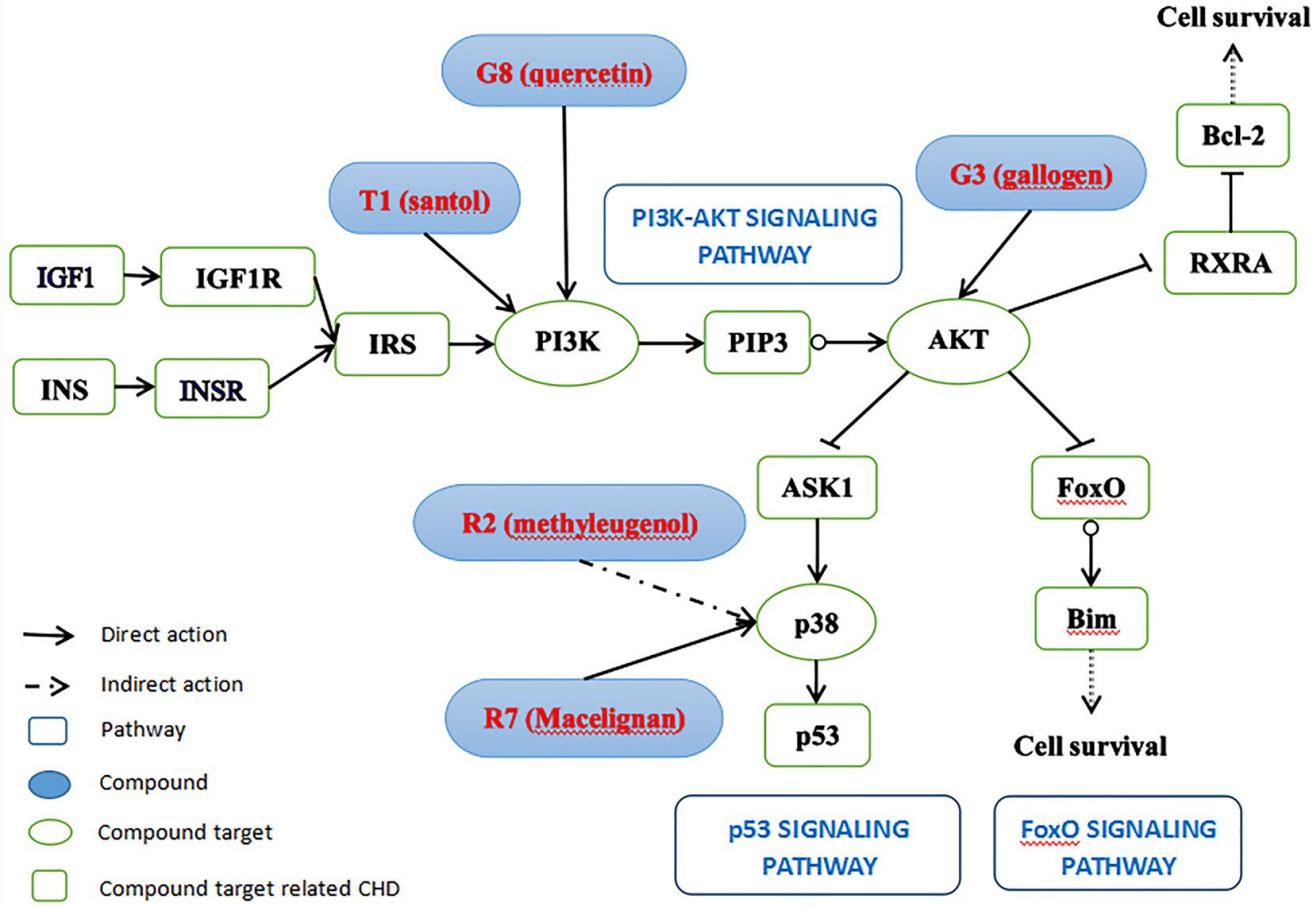
The relation between G3, G8, R2, R7, T1 and the regulated protein.

Our previous bioinformatics study profiled the molecular mechanisms of the herbal prescription SWTX against CHD through multi-level data integration, the multiple ingredients derived from SWTX may regulate many aspects of CHD, such as stress response, angiogenesis, immune inflammatory response, energy metabolism, endocrine regulation, etc., and the PI3K/Akt/FoxO3a pathway was identified to be one of the most correlated pathways^8^. The present experiment study further validates that the involvement of the PI3K/Akt/FoxO3a pathway is an important step for SWTX against myocardial ischemia reperfusion injury in the rat I/R model. In vivo experiments, SWTX can effectively inhibit I/R injury in rats by reducing ST-segment elevation, pathological changes and myocardial infarct size and improving cardiac function, while SWTX up-regulate *Pik3r1*, *Akt,* down-regulate *Bim* gene, and inhibits the reduction of Akt1, FoxO3a phosphorylation mediated by I/R injury. Three herbs, GZ, RDK and TX, had synergistic effects. In vitro experiments,gallogen (G3) and quercetin (G8) of GZ, methyleugenol (R2) and macelignan (R7) of RDK, santol (T1) of TX are identified as active phytochemicals in SWTX that suppressed the injury of H_2_O_2_. Quercetin (G8) and santol (T1) reverse p-Akt1 repression induced by H_2_O_2_, but R2 and R7 have no significant effect, while four monomers G8, R2, R7 and T1 promote phosphorylation of FoxO3a to decrease the FoxO3a translocation and inhibit apoptosis. It’s worth noting that, Bim triggers mitochondrial apoptosis, the relation between G3, G8, R2, R7, T1 and the regulated protein were indicated in the Fig. 7. However, both in vivo and in vitro, single Bim expression in the model groups were reduced or stayed the same rather than increased, while Bim in drug intervention groups exhibited a marked increasing, PI3K inhibitor LY294002 totally or partly reversed the above effects of G8, R7, R5 and T1. It is reason that the increasing of single Bim expression results in suppression of the Bim–DLC1–Mcl-1 complexes with stronger pro-apoptotic activity. The *Bim* gene was up-regulated by I/R injury in vivo, and was efficiently reversed by GZ, GZ + RDK and SWTX, SWTX has highest advantages. It has been reported that Bim bound anti-apoptotic Mcl-1 with the participation of dynein light chain 1 (DLC1) to form the large complexes (Bim-DLC1-Mcl-1) at the mitochondrial outer membrane, meanwhile Bim complexes were more active at binding anti-apoptotic Bcl-2 proteins than noncomplexed Bim, it has been confirmed that this large complexes has a higher proapoptotic activity than wild-type Bim (single Bim) in all cell lines that they tested, although in Bim-dependent apoptosis. In present results, Bim protein expression was significantly reduced by I/R injury in vivo model or H2O2-induced injury in vitro compared with the control group. Therefore, it is reasonable to think that these injury facilitated formation of the large complexes with higher pro-apoptotic activity. The treatment of monomers of SWTX in vitro or GZ, GZ + RDK or SWTX in vivo could inhibit the formation of the large complexes (Bim-DLC1-Mcl-1) by down-regulating the wild-type Bim, therefore the relative expression of wild-type Bim manifest as a state of up-regulating, comparing with the model group.

Thus, these results suggest that SWTX decreases I/R injury, and the PI3K/Akt/FoxO3a pathway takes part in protection during this process, gallogen (G3) and quercetin (G8) of GZ, methyleugenol (R2) and macelignan (R7) of RDK, santol (T1) of TX are responsible at least in part for SWTX’s cardioprotection effect. This study can only uncover a little pharmacological action of SWTX against CHD. At present, we do not know the molecular basis and active ingredients of SWTX on angiogenesis, chronic inflammatory response, endocrine regulation and other factors closely related to CHD. The future study will need to address these questions.

### Data analysis

All experiments were repeated at least 3 times and presented as mean ± SD and analyzed by one-way ANOVA. *P* < 0.05 was considered to indicate a statistically significant difference. All tests were performed using GraphPad Prism 7 software (GraphPad Software, Inc., La Jolla, CA, USA).

## Supplementary Information

Supplementary Information

## Data Availability

The datasets generated during and/or analyzed in the current study are available from the corresponding author upon request.
