# Author Correction: Validation of efficacy and mechanism of Sanwei-Tanxiang powder in improving myocardial ischemia reperfusion injuries

**DOI:** 10.1038/s41598-021-92884-8

**Published:** 2021-06-22

**Authors:** Yu‑Hui Sun, Ren Bu, Yue‑Wu Wang, Yu‑Chong Hu, Xu‑Mei Wang, Xin Dong, Wen Zu, Yan Niu, Peng‑Wei Zhao, Peng Sun, Shi‑Hang Ru, Jing‑Kun Lu, Sheng‑Sang Na

**Affiliations:** 1 Department of Pharmacy, Chifeng Municipal Hospital, Chifeng, China; 2 School of Pharmacy, Inner Mongolia Medical University, Huhehot, China; 3 Center for New Drug Safety Evaluation and Research, Inner Mongolia Medical University, Huhehot, China; 4 Inner Mongolia Autonomous Region People’s Hospital, Huhehot, China; 5 Inner Mongolia Medical University, Huhehot, China; 6 School of Basic Medicine, Inner Mongolia Medical University, Huhehot, China; 7 Radiotherapy Department, Chifeng Municipal Hospital, Chifeng, China; 8 Institute of Mongolian Medicine, Inner Mongolia Medical University, Huhehot, China

Correction to: *Scientific Reports* 10.1038/s41598-020-80861-6, published online 12 January 2021

This Article contains an error in the legend of Figure 1, where,

“(D) The level of LDH. (E) The level of CK.”

should read:

“(D) The level of CK. (E) The level of LDH.”

