# Supplementary material for: Validation of efficacy and mechanism of Sanwei-Tanxiang powder in improving myocardial ischemia reperfusion injuries
Source: Sci Rep. 2021 Jan 12;11:664. doi: 10.1038/s41598-020-80861-6 (PMC7804470; doi:10.1038/s41598-020-80861-6)
Supplement: Supplementary file 1 — Supplementary Information [file 41598_2020_80861_MOESM1_ESM.docx]

**Validation of efficacy and mechanism of Sanwei-Tanxiang powder in improving myocardial ischemia reperfusion injuries**

**Yu-Hui Sun^1#^, Ren Bu^2#^,****Yue-Wu Wang^3^, Yu-Chong Hu^4^, Xu-Mei Wang^1^, Xin Dong^2^, Wen Zu^5^, Yan Niu^6^, Peng-Wei Zhao^6^, Peng Sun^6^, Shi-Hang Ru^7^, Jing-Kun Lu^6＊^&Sheng-Sang Na^8＊^**

^1^Department of Pharmacy, Chifeng Municipal Hospital, Chifeng, China.

^2^School of Pharmacy, Inner Mongolia Medical University, Huhehot, China.

^3^Center for New Drug Safety Evaluation and Research, Inner Mongolia Medical University, Huhehot, China.

^4^Inner Mongolia Autonomous Region People’s Hospital. Huhehot, China.

^5^Library, Inner Mongolia Medical University, Huhehot, China.

^6^School of Basic Medicine, Inner Mongolia Medical University, Huhehot, China.

^7^Radiotherapy Department, Affiliated Hospital of Chifeng University, Chifeng, China.

^8^Institute of Mongolian Medicine, Inner Mongolia Medical University, Huhehot, China.





Supplementary Figure S1: GZ, GZ+RDK, SWTX administration didn’t disturb the basic cardiac physiological parameters.





Supplementary Figure S2: Effects of the activemonomers of SWTX on H_2_O_2_-induced H9c2 cell oxidative injury. (A) 12.5, 50, 200μΜ G1, G2, G3, G4, G5, G6, G8, R1, R2, R3, R4, R5, R6, R7 and T1 pretreated H9c2 cell for 2h and co-incubated with 900μΜ H_2_O_2_ for additional 22h, to observe the H9c2 cell viability in comparison to the cells singly treated with 900μΜ H_2_O_2_ (*n*=6). (B) 12.5, 50, 200μΜ G3, G8, R3, R5 and T1 pretreated H9c2 cell for 2h and co-incubated with 900μΜ H_2_O_2_ for additional 22h, significantly improved H9c2 cell viability in comparison to the cells singly treated with 900μΜ H_2_O_2_, among them, G8 and T1 shown best (n=6).


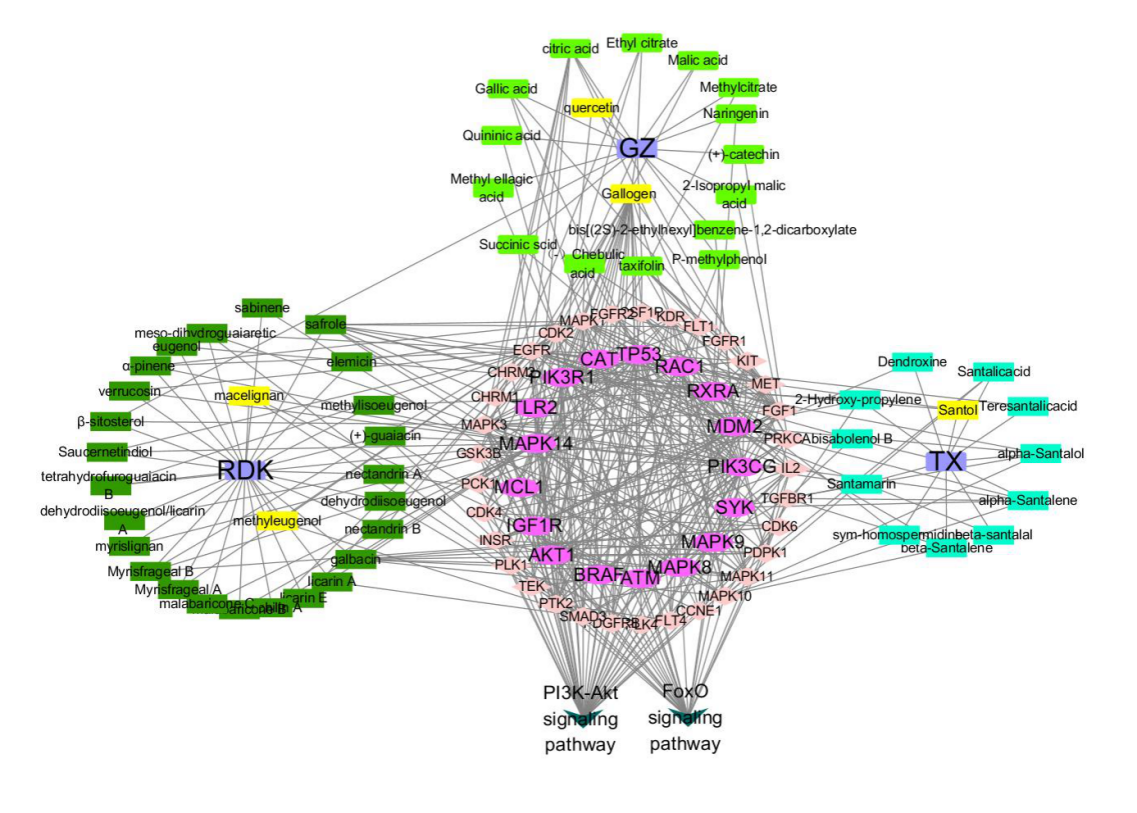


Supplementary Figure S3：Major components of GZ, RDK and TX, which are related to the PI3K/Akt/FoxO3a pathway.


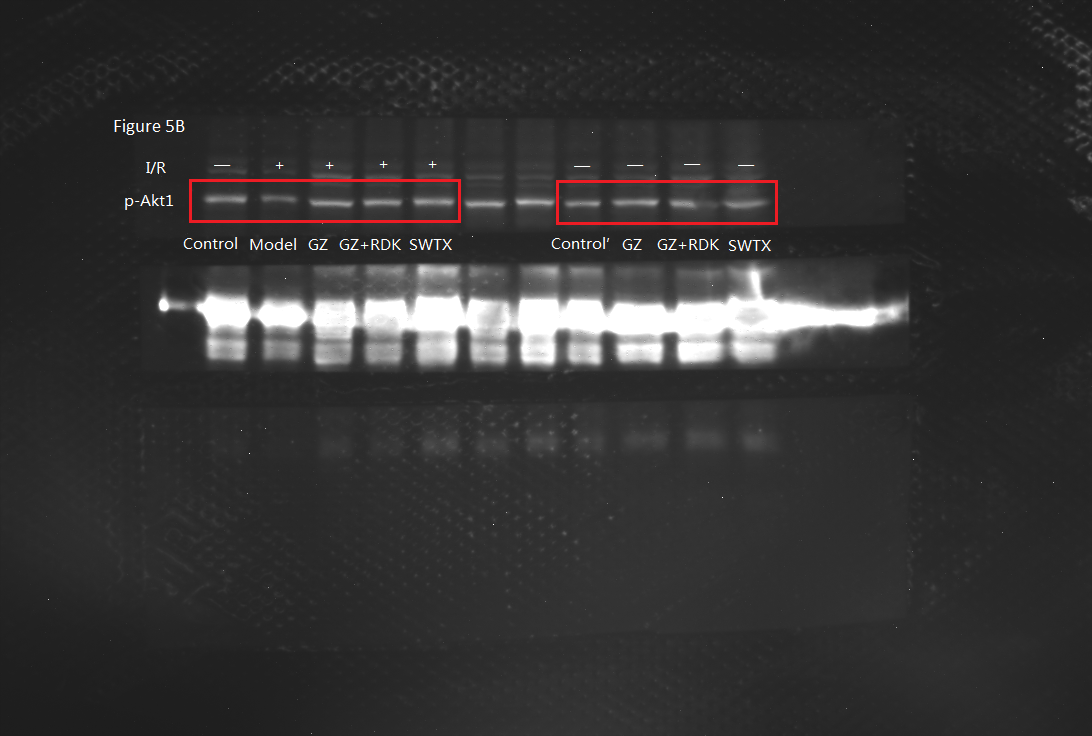


Supplementary Figure S4: The representative Western blots shown the effects of SWTX on the expression of p-Akt1 (Figure 5B).


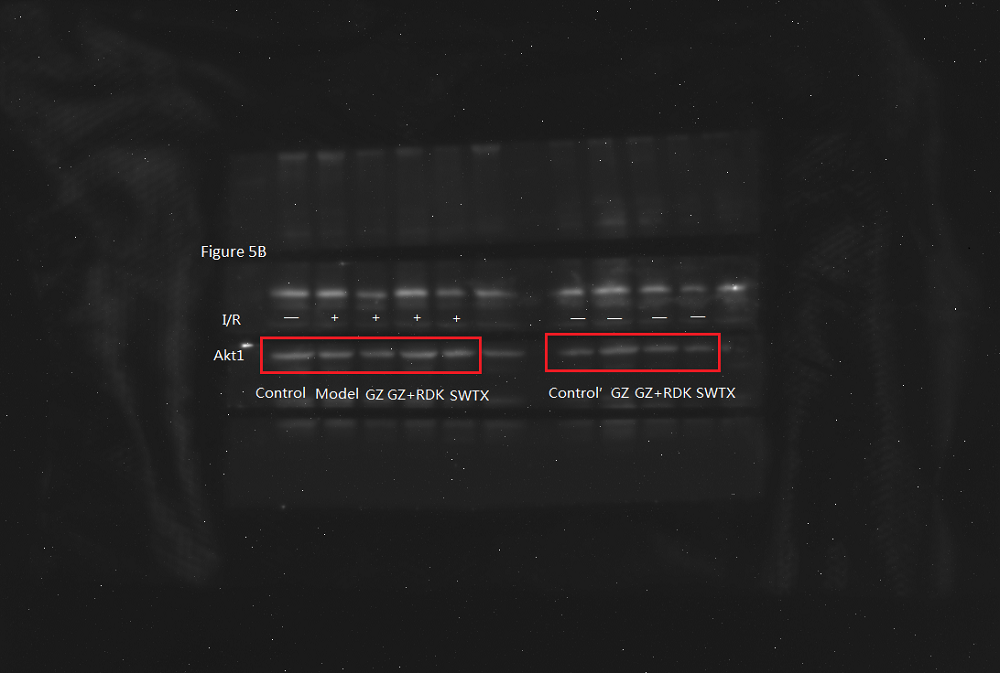


Supplementary Figure S5: The representative Western blots shown the effects of SWTX on the expression of Akt1 (Figure 5B).


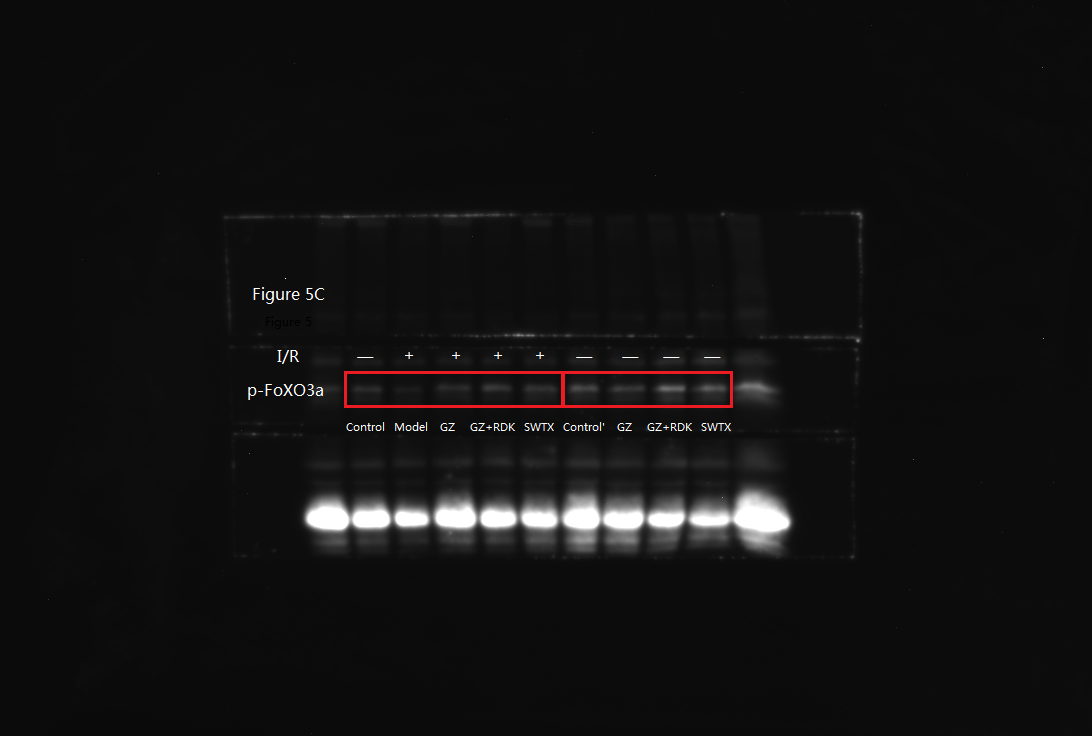


Supplementary Figure S6: The representative Western blots shown the effects of SWTX on the expression of p-FoxO3a (Figure 5C).


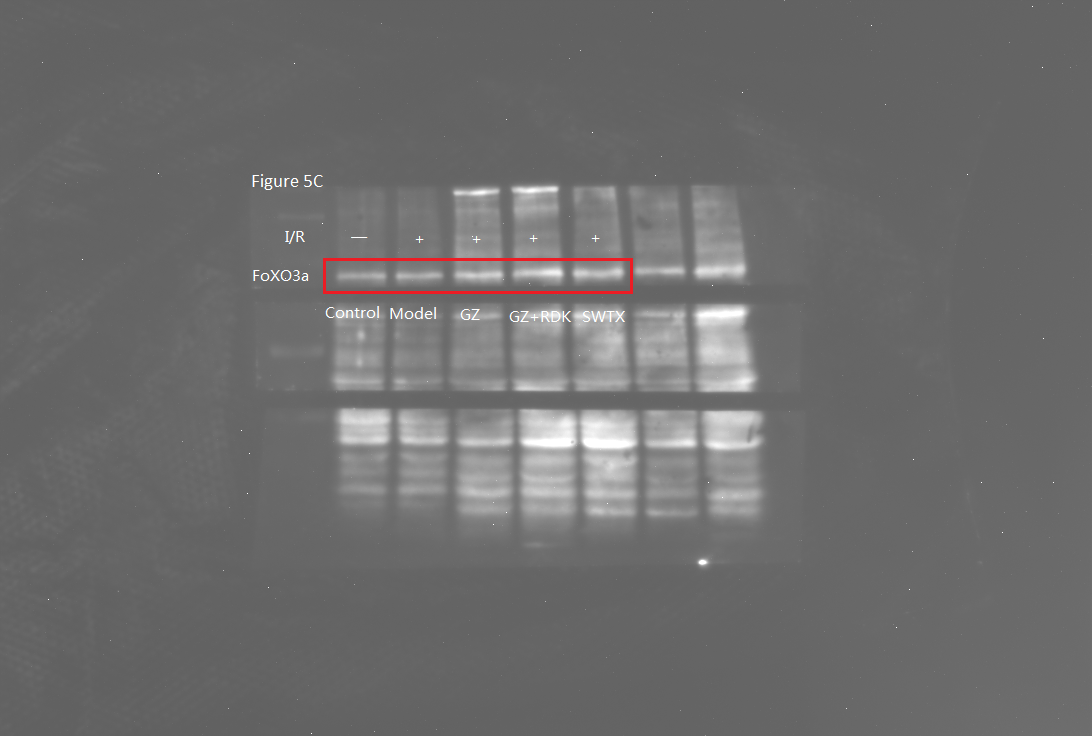


Supplementary Figure S7: The representative Western blots shown the effects of SWTX on the expression of FoxO3a (Figure 5C: Control, Model, GZ, GZ+RDK, SWTX).


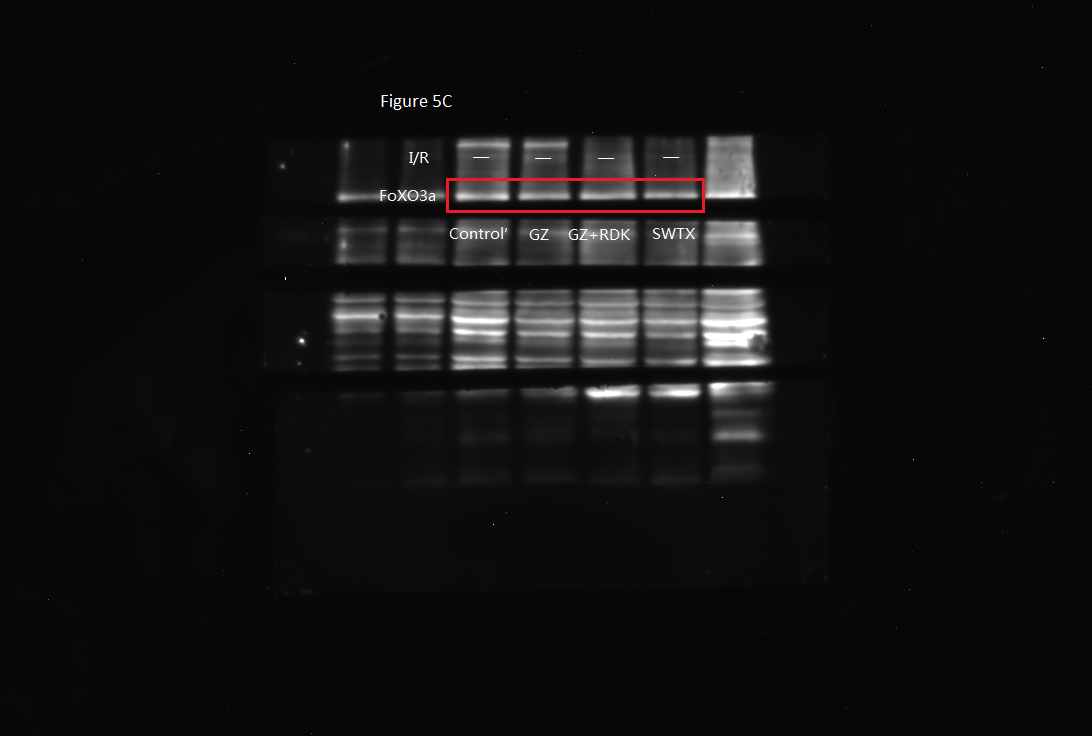


Supplementary Figure S8: The representative Western blots shown the effects of SWTX on the expression of FoxO3a (Figure 5C: Control’, GZ, GZ+RDK, SWTX).


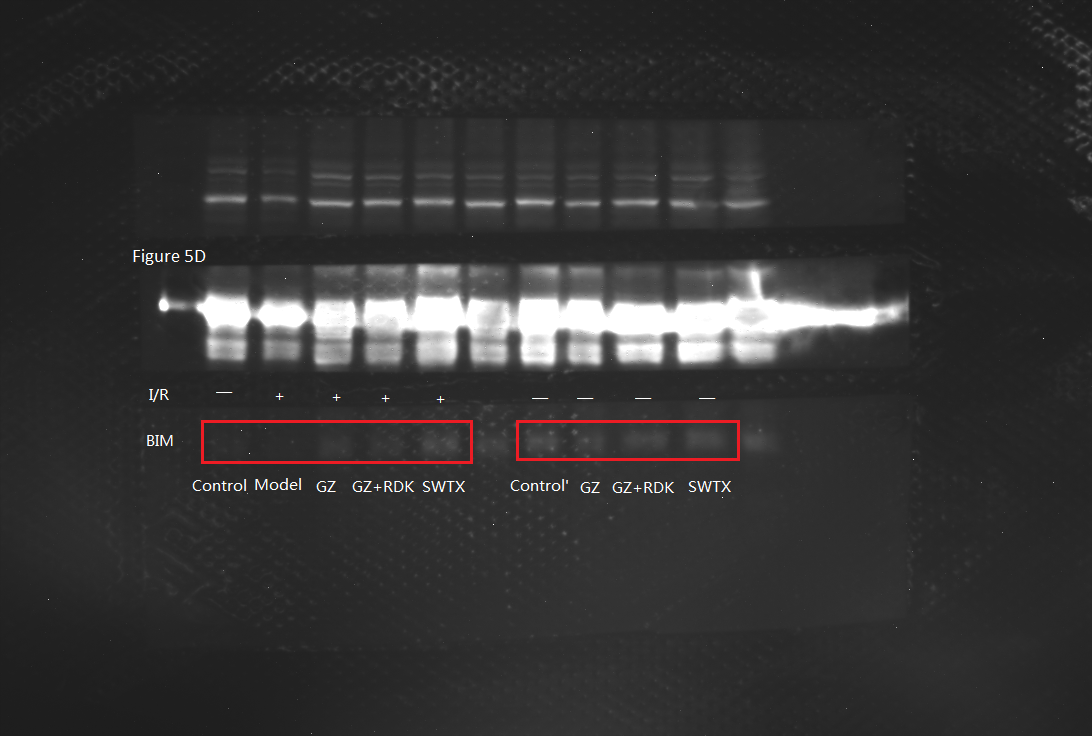


Supplementary Figure S9: The representative Western blots shown the effects of SWTX on the expression of BIM (Figure 5D).


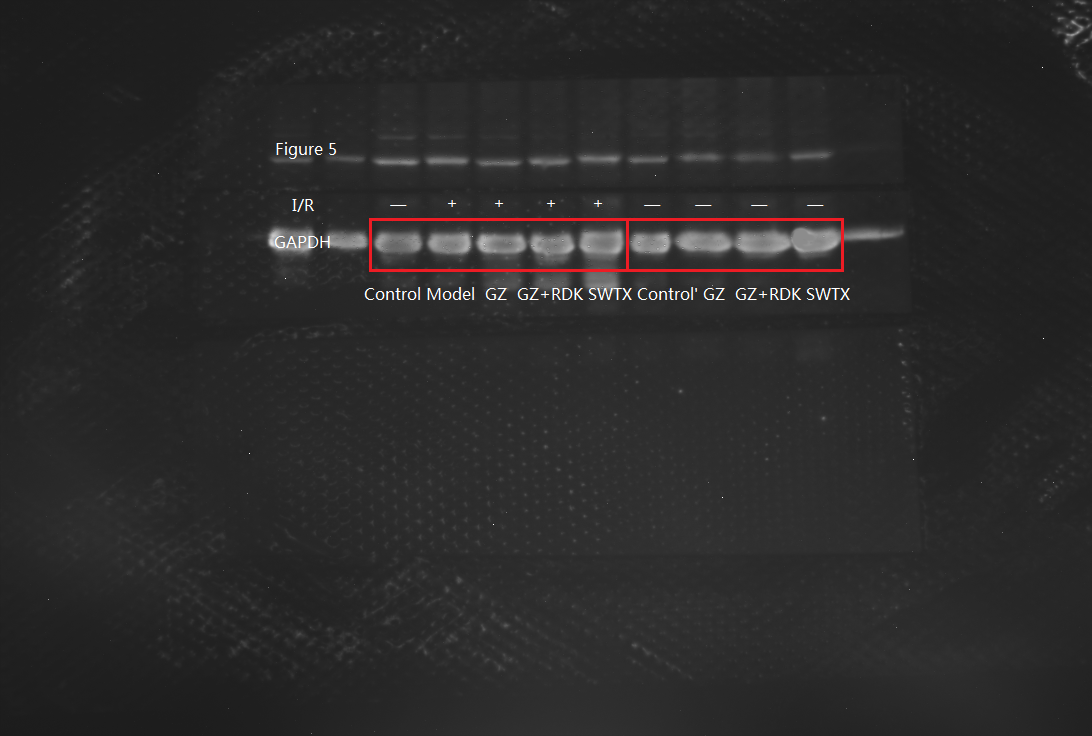


Supplementary Figure S10: GAPDH was used as loading control (Figure 5).


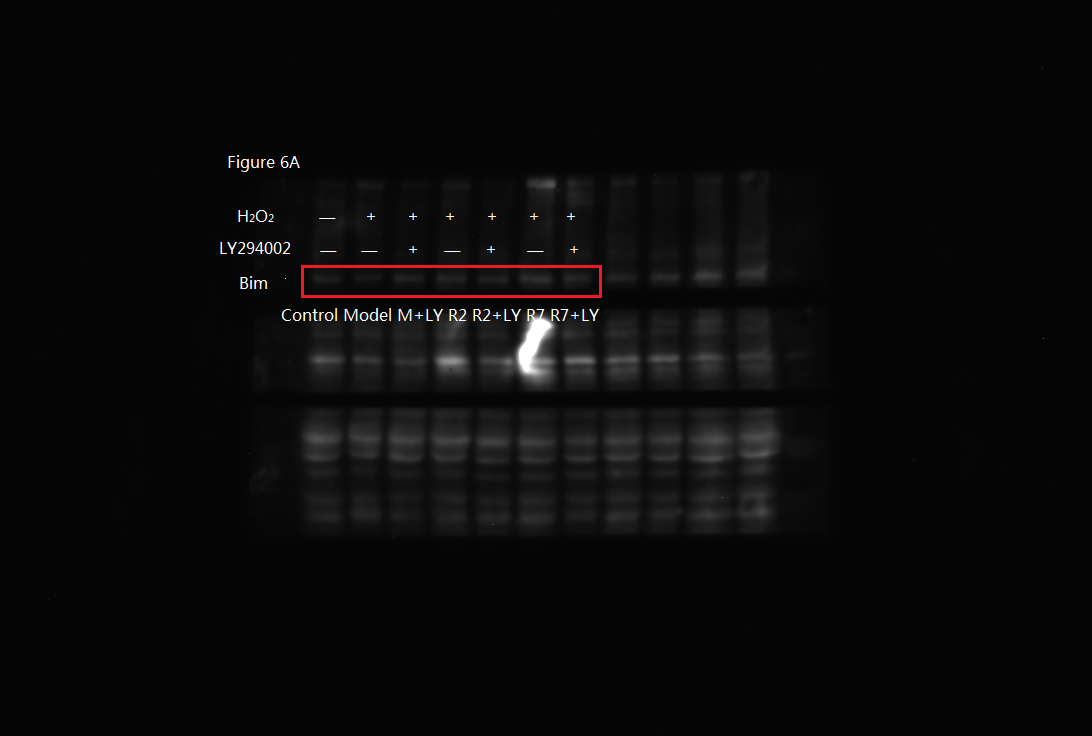


Supplementary Figure S11: The representative Western blots shown the effects of R2, R7 on the expression of Bim and the interfering effects of LY294002 (LY) (Figure 6A).


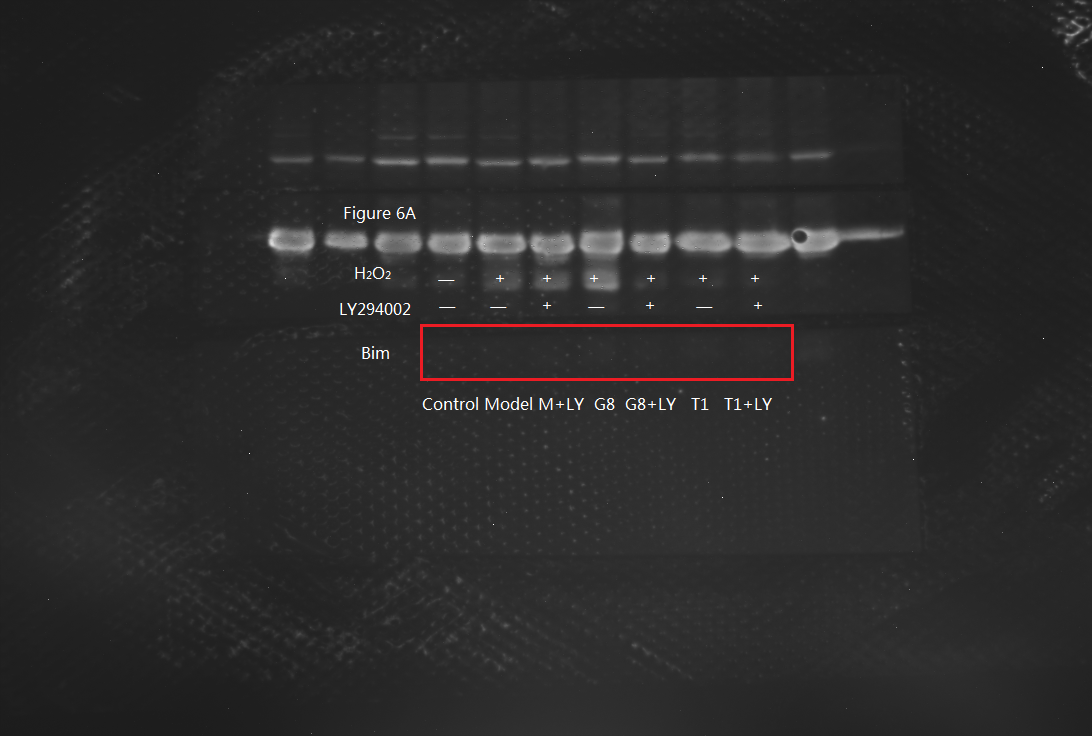


Supplementary Figure S12: The representative Western blots shown the effects of G8, T1 on the expression of Bim and the interfering effects of LY294002 (LY) (Figure 6A).


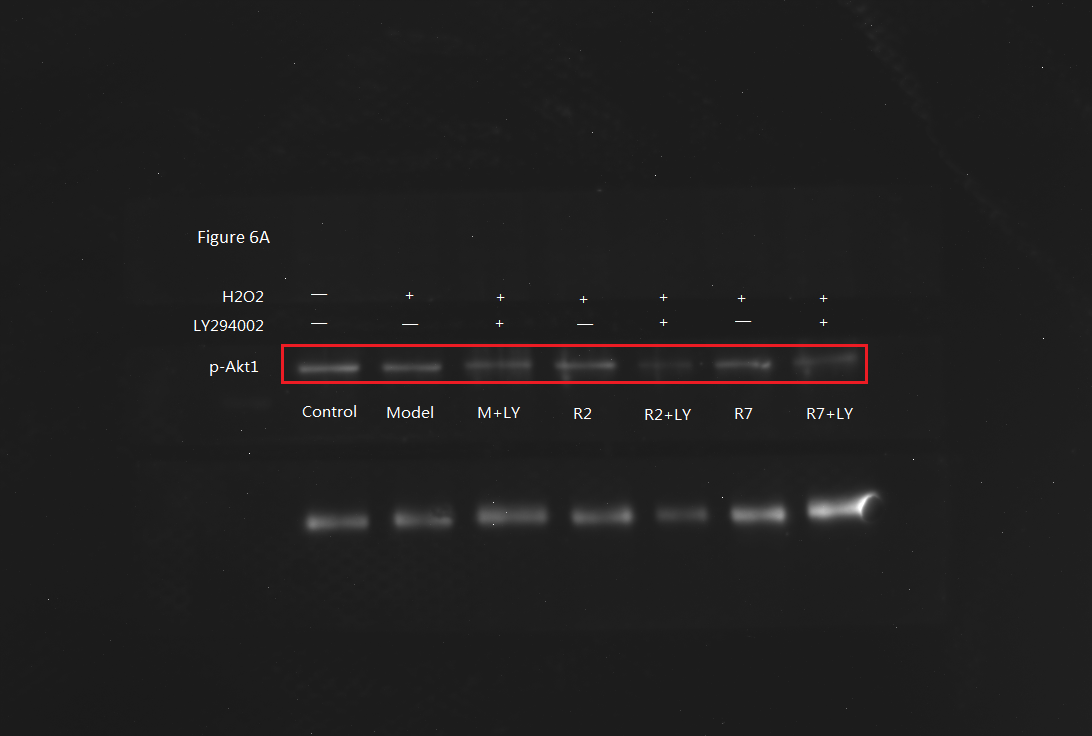


Supplementary Figure S13: The representative Western blots shown the effects of R2, R7 on the expression of p-Akt1 and the interfering effects of LY294002 (LY) (Figure 6A).

**
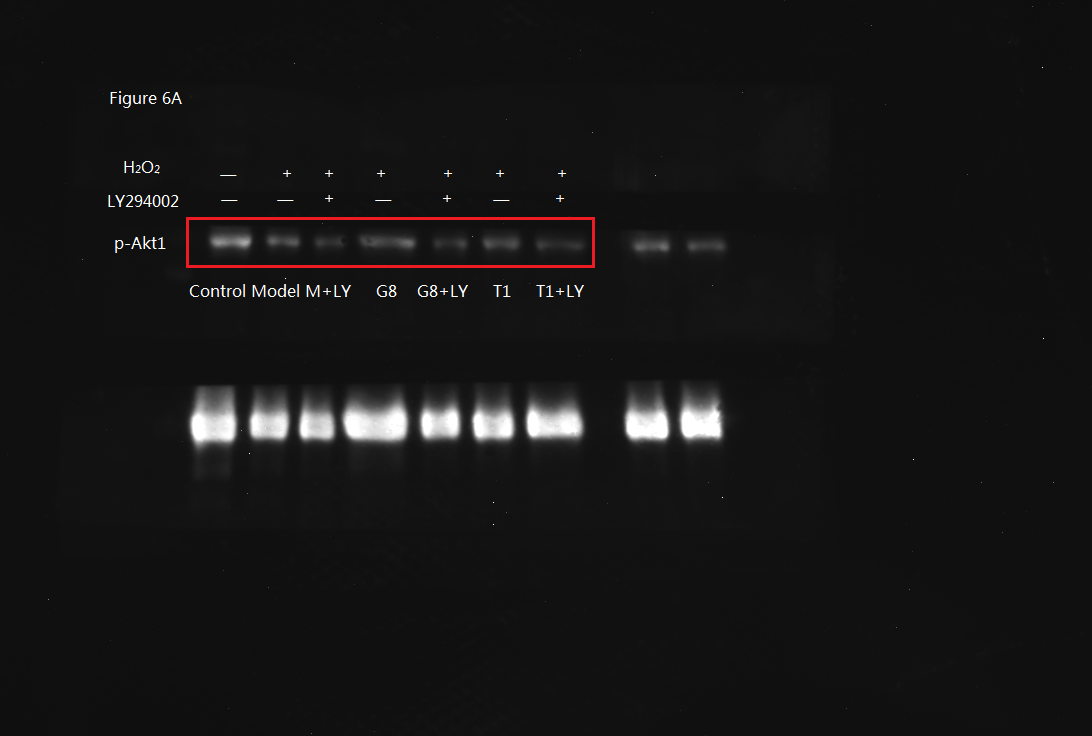
**

Supplementary Figure S14: The representative Western blots shown the effects of G8,T1 on the expression of p-Akt1 and the interfering effects of LY294002 (LY) (Figure 6A).

**
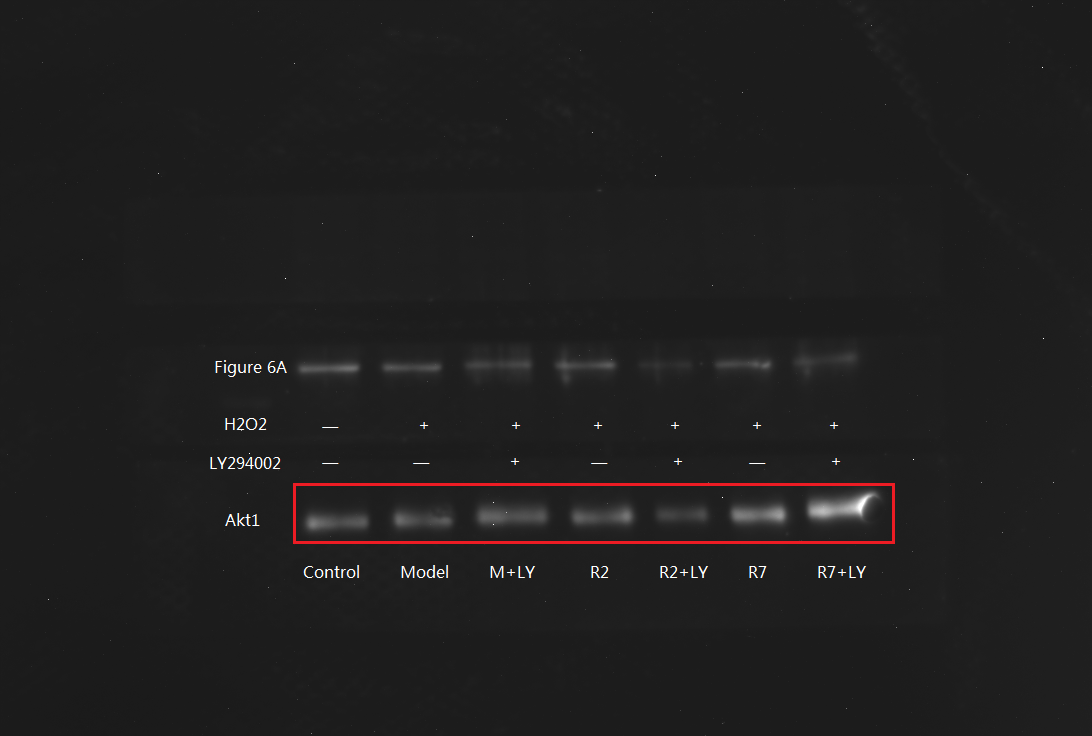
**

Supplementary Figure S15: The representative Western blots shown the effects of R2, R7 on the expression of Akt1 and the interfering effects of LY294002 (LY) (Figure 6A).

**
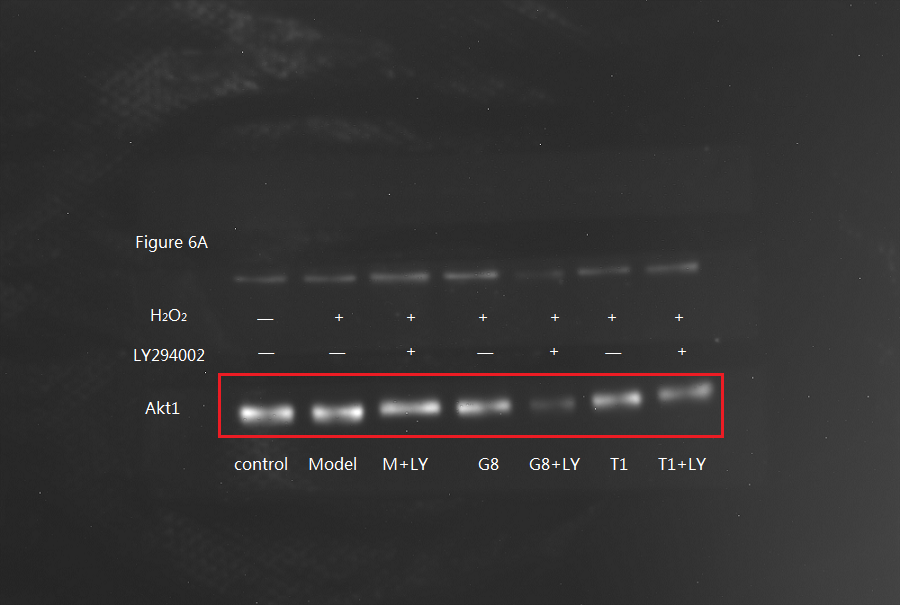
**

Supplementary Figure S16: The representative Western blots shown the effects of G8, T1 on the expression of Akt1 and the interfering effects of LY294002 (LY) (Figure 6A).

**
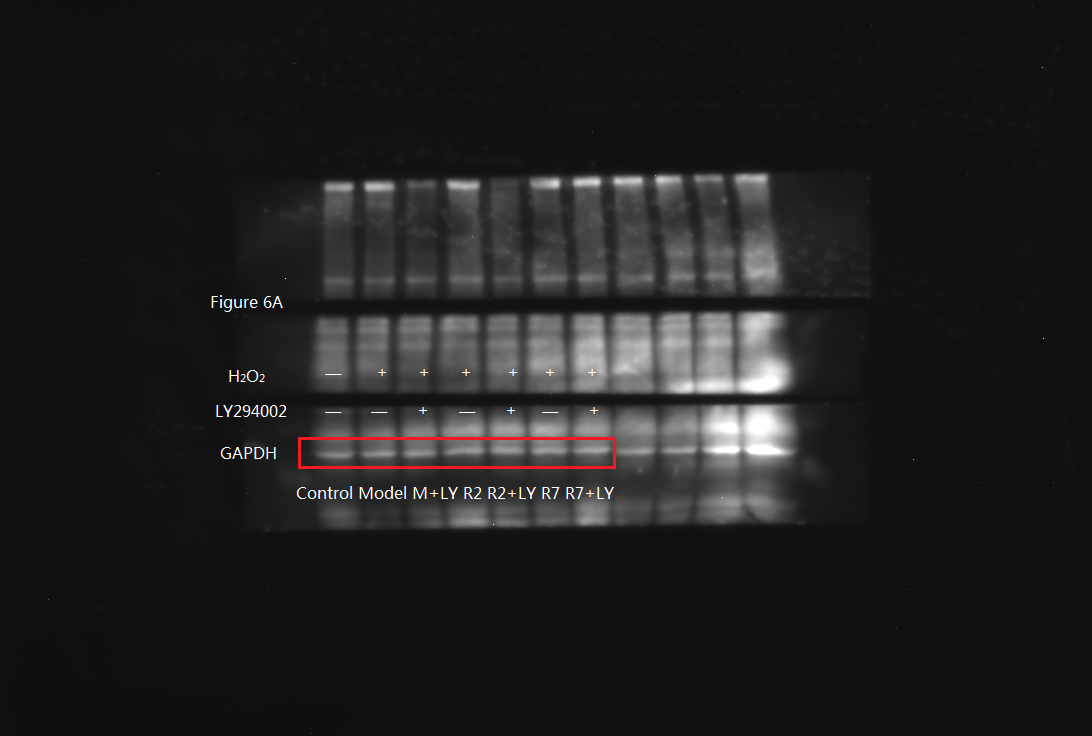
**

Supplementary Figure S17: GAPDH was used as loading control of R2, R7 (Figure 6A).


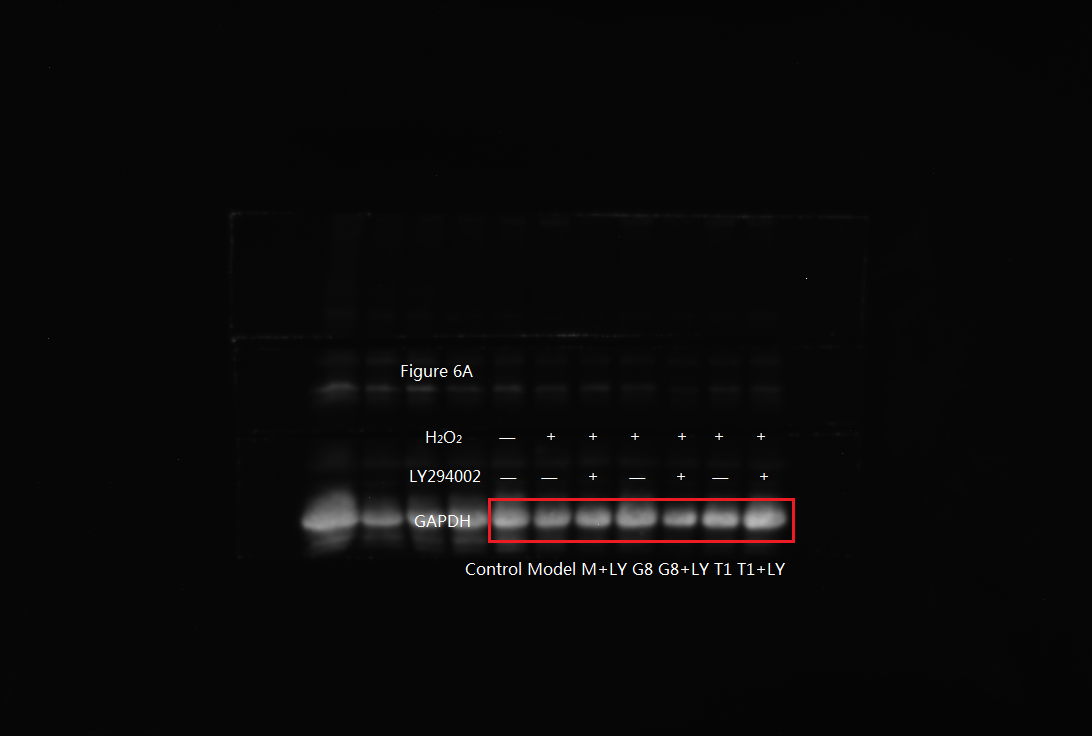


Supplementary Figure S18: GAPDH was used as loading control of G8, T1 (Figure 6A).

**Supplementary Table S1** The predicted active monomers of SWTX

| Herbs | Code | Active monomers | Chemical structure | Basis |
| --- | --- | --- | --- | --- |
| GZ | G1 | （Kaempferol） |  | Into the blood components |
|  | G2 | (citric acid) |  | Into the blood components |
|  | G3 | (gallogen/ellagic acid) |  | Into the blood components |
|  | G4 | (gallic acid) |  | Into the blood components |
|  | G5 | (succinic acid) |  | Into the blood components |
|  | G6 | (+)-catechin |  | Into the blood components |
|  | G8 | (quercetin) |  | Into the blood components |
| RDK | R1 | (eugenol) |  | Content is higher |
|  | R2 | (methyleugenol) |  | Content is higher |
|  | R3 | (methylisoeugenol) |  | Content is higher |
|  | R4 | (myristicin) |  | Content is higher, toxic, Antimicrobial properties, Antioxidant activity, Into the blood components |
|  | R5 | (elemicin) |  | Content is higher |
|  | R6 | （Licarin B） |  | Content is higher |
|  | R7 | (macelignan) |  | The anti-inflammatory, to suppress AMPK, and pharmacological activity of protecting liver |
| TX | T1 | (santol) |  | Content is higher |

**Supplementary Table S2** The active monomers of SWTX exerted effect against H_2_O_2_-induced oxidative injury on H9c2 cells. (*$\overline{\text{x}}$*±s，n=4)

| Group | Concentration (μmol·L^-1^) | Cell survival rate (%) |
| --- | --- | --- |
| Contrast | -- | 100.00±3.20 |
|  |  |  |
|  |  |  |
| H/R | -- | 43.05±5.35^##^ |
|  |  |  |
|  |  |  |
| H/R+G1 | 200 | 32.68±1.52* |
|  | 50 | 61.08±3.04** |
|  | 12.5 | 24.87±1.42** |
| H/R+G2 | 200 | 61.20±1.21** |
|  | 50 | 61.78±3.58** |
|  | 12.5 | 45.27±1.52** |
| H/R+G3 | 200 | 39.73±1.87 |
|  | 50 | 70.07±5.65** |
|  | 12.5 | 65.28±5.32** |
| H/R+G4 | 200 | 33.32±3.31* |
|  | 50 | 25.66±1.68** |
|  | 12.5 | 27.44±2.71 |
| H/R+G5 | 200 | 20.37±0.76** |
|  | 50 | 50.91±1.15 |
|  | 12.5 | 50.71±1.78 |
| H/R+G6 | 200 | 50.10±0.66** |
|  | 50 | 47.03±1.78 |
|  | 12.5 | 45.33±2.92 |
| H/R+G8 | 200 | 51.23±5.67 |
|  | 50 | 100.99±4.91** |
|  | 12.5 | 94.68±2.42** |
| H/R+R1 | 200 | 35.99±14.02 |
|  | 50 | 45.19±4.85 |
|  | 12.5 | 32.49±7.00** |
| H/R+R2 | 200 | 52.65±4.11 |
|  | 50 | 76.82±4.78** |
|  | 12.5 | 47.04±2.14 |
| H/R+R3 | 200 | 39.73±1.87 |
|  | 50 | 70.07±5.65** |
|  | 12.5 | 65.28±5.32** |
| H/R+R4 | 200 | 54.37±6.49* |
|  | 50 | 70.90±5.79** |
|  | 12.5 | 24.83±1.28** |
| H/R+R5 | 200 | 52.32±2.27 |
|  | 50 | 59.83±4.26** |
|  | 12.5 | 36.40±4.08 |
| H/R+R6 | 200 | 19.12±1.59** |
|  | 50 | 51.35±1.12 |
|  | 12.5 | 47.92±3.99 |
| H/R+R7 | 200 | 54.37±6.49* |
|  | 50 | 74.57±2.44** |
|  | 12.5 | 24.83±1.28** |
| H/R+T1 | 200 | 82.93±4.77** |
|  | 50 | 103.85±8.00** |
|  | 12.5 | 85.07±3.84** |
